# Overexpression of a Novel Cytochrome P450 Promotes Flavonoid Biosynthesis and Osmotic Stress Tolerance in Transgenic *Arabidopsis*

**DOI:** 10.3390/genes10100756

**Published:** 2019-09-26

**Authors:** Naveed Ahmad, Liu Jianyu, Tian Xu, Muhammad Noman, Aysha Jameel, Yao Na, Dong Yuanyuan, Wang Nan, Li Xiaowei, Wang Fawei, Liu Xiuming, Li Haiyan

**Affiliations:** 1 Ministry of Education Engineering Research Center of Bioreactor and Pharmaceutical Development, Jilin Agricultural University, Changchun 130118, China; naveedjlau@gmail.com (N.A.);; 2 College of Life Sciences, Jilin Agricultural University, Changchun 130118, China

**Keywords:** cytochrome P450, heterologous expression, transgenic *Arabidopsis*, abiotic stress, flavonoid biosynthesis

## Abstract

Flavonoids are mainly associated with growth, development, and responses to diverse abiotic stresses in plants. A growing amount of data have demonstrated the biosynthesis of flavonoids through multienzyme complexes of which the membrane-bounded cytochrome P450 supergene family shares a crucial part. However, the explicit regulation mechanism of Cytochrome P450s related to flavonoid biosynthesis largely remains elusive. In the present study, we reported the identification of a stress-tolerant flavonoid biosynthetic *CtCYP82G24* gene from *Carthamus tinctorius*. The transient transformation of *CtCYP82G24* determined the subcellular localization to the cytosol. Heterologously expressed *CtCYP82G24* was effective to catalyze the substrate-specific conversion, promoting the de novo biosynthesis of flavonoids in vitro. Furthermore, a qRT-PCR assay and the accumulation of metabolites demonstrated that the expression of *CtCYP82G24* was effectively induced by Polyethylene glycol stress in transgenic *Arabidopsis*. In addition, the overexpression of *CtCYP82G24* could also trigger expression levels of several other flavonoid biosynthetic genes in transgenic plants. Taken together, our findings suggest that *CtCYP82G24* overexpression plays a decisive regulatory role in PEG-induced osmotic stress tolerance and alleviates flavonoid accumulation in transgenic *Arabidopsis*.

## 1. Introduction

During natural flavonoid biosynthesis, chalcone synthase (CHS) utilizes phenylpropanoids and malonyl-CoA as general substrates to catalyze the phenylpropanoid pathway in plants. Following other metabolic routes, these precursor compounds can be converted into six different subclasses including flavonols, anthocyanins, flavones, and flavan-3-ols, the last of which includes proanthocyanidins, catechins, phenolic acids and flavonols [1]. Various groups of multienzyme complexes are known to catalyze the biochemical reactions of plant physiological pathways and their adaptation to ever-changing environments [2,3]. These complexes of enzymes are mainly located in the cytosol and also found embedded near the inner side of the endoplasmic reticulum (ER). The ER-bound enzyme complex belongs to the cytochrome P450 superfamily [4,5]. In most of the plant species, cytochrome P450 is found abundantly and produces multiple groups of secondary metabolites through hydroxylation and monooxygenation reactions. CYP701, a member of cytochrome P450 encoding ent-kaurene oxidase, is essential for the biosynthesis of gibberellins [6,7]. Recent studies have reported the characterization of CYP90A/B and CYP724, CYP93, CYP73A, CYP75A, and CYP81 as cinnamate 4-hydroxylase, flavonoid 3′,5′-hydroxylase, flavonoid 3′-hydroxylase and flavone synthase II, respectively, to catalyze the oxidative reactions during brassinosteroid production and flavonoid biosynthesis in plants [8,9,10]. Furthermore, several other studies have discovered the pharmacological importance of the cytochrome P450 supergene family by stimulating plant immunity against various pathogens [11,12]. Recently, scientists have discovered that anthocyanins could minimize the risk of many chronic diseases like hypertension [13] dysentery, diarrhoea and cardiovascular diseases [14,15].

Safflower (*Carthamus tinctorius* L.) also known as ‘Bastard saffrons’’ is a highly broadleaf plant belongs to the Asteraceae family. Due to the increasing demand for safflower oilseed, which highly rich in conjugated linoleic acid, it has attracted the attention of plant biologists worldwide. Safflower’s oilseed consists of 80% of octadecadienoic acid, which can regulate the rate of cholesterol and avert diseases related to cardiovascular channels [16]. Over 5000 types of phenolic compounds exist across the plant kingdom in which safflower shares a remarkable variety of flavonoids including carthamin chalcone glycoside, kaempferol glucosides, hydroxy safflor yellow A and B, and quercetin glucosides. [17,18]. Therefore, safflower is radically recommended for its medicinal and economic value in mainland of China and West Asia. Though the diversity of flavonoid biosynthesis has been reported in *Arabidopsis thaliana* seeds [19], the mechanism of stress-responsive cytochrome P450 genes encoding a single or group of enzymes promoting the flavonoid biosynthesis pathway in *Arabidopsis* remains unexplained. As a point of paramount significance, it is important to focus on studies related to identification and characterization of putative cytochrome P450 genes promoting the flavonoid biosynthetic pathway in plants. In the present study, we report the discovery and characterization of a 1368bp long fragment of cDNA which encodes a novel CYP450 (CtCYP82G24) from *Carthamus tinctorius* using an expressed sequence, homology-based approach followed by green fluorescent protein tagging and expression analysis in a transgenic system. In addition, comparative analyses of qRT-PCR assay and metabolite accumulation were carried out to examine the mechanism of PEG-induced osmotic stress tolerance in CtCYP82G24 overexpressed plants by upregulating the transcription of multiple flavonoid pathway genes. Our results provide adequate information and novel insight to understand the regulatory mechanism of a new osmotic stress-responsive CYP gene in transgenic *Arabidopsis*.

## 2. Materials and Methods

### 2.1. Plant Materials, Vectors, and Strains

*Carthamus tinctorius* cultivar, *Jihong No.1* seeds were purchased from Fuyu Seeds Company, China. Wild-type *A. thaliana* was grown for 5–7 weeks, and the early flowering plants were subjected to floral-dip transformation. *Agrobacterium tumefacien* strain EHA105, *Escherichia coli* BL21, *E. coli* DH5α, the prokaryotic expression vector (PET28a-CtCYP82G24), (the plant over-expression vector pBASTA-CtCYP82G24), the subcellular localization vector (CtCYP82G24-pCAMBIA1302-GFP (green fluorescent protein) was successfully constructed and preserved the 80% glycerol stock at −80 °C until next use. Restriction enzymes (BamH1, EcoR1, BglII, and Spe1) and DNA ligase (T4) were purchased from Takara Biotechnology Company Beijing, China.

### 2.2. De Novo Transcriptomic Assembly

A total of 454 sequencing libraries were generated from the total RNA samples of the early and full flowering stages of *Carthamus tinctorius* using a GS-FLX sequencer (Roche, Basel, Switzerland). Clean reads were obtained, were followed by the deletion the raw data including low-quality reads, adapter reads, hairpin structural reads and shorter reads (<50 bp), and were then assembled into unigenes using the MIRA program [20]. The longest transcripts were further selected as unigenes for functional analysis by identifying their corresponding nucleotide sequences. The study of functional annotations and pathway enrichment analysis of the unigenes were investigated using a non-redundant protein sequence (http://www.ncbi.nlm.nih.gov) and non-redundant nucleotide sequences (http://www.ncbi.nlm.nih.gov/). Further annotations were analyzed in SwissProt. Gene ontology (GO) annotations were carried out using blast2GO (http://www.geneontology.org/) [21]. To determine the high-level functional enrichment of biosynthetic systems from molecular-level information, the Kyoto Encyclopedia of Genes and Genomes pathways analysis was performed using GenMAPP 2.1 (http://www.genmapp.org/).

### 2.3. Characterization and Phylogenetic Analysis

Based on the information above, the multiple sequence alignment of the candidate *CtCYP82G24* was built using the DNAMAN software (Lynnon Corp., Quebec, Canada) and MEGA. v5 by implementing auto-reorder tools [22]. The occurrence of gaps in the alignment results was removed, followed by a 1000 rapid bootstraps method. The resulted phylogenetic tree was edited and visualized using MEGA v5. The number of the open reading frame was calculated using Laser gene SeqMan II Module DNAStar (www.DNAstar.com). The three-dimensional protein model was created by implementing Protein Homology/analogy Recognition Engine V 2.0 Phyre^2^ software [23]. The expected amino acid sequence, isoelectric point, and molecular weight of *CtCYP82G24* were identified with the help of ExPASy ProtParam software (http://web.expasy.org/protparam/).

The complete coding sequence of *CtCYP82G24* was deposited in GenBank under accession number MK583014. In addition, the conserved motifs of the CtCYP82G24 protein were screened and analyzed with the help of the online web tool Multiple EM for Motif Elicitation version 4.8.1 (http://meme-suite.org/tools/meme). The following parameter settings were used: The per sequence occurrence of the single motif was optimized from 2 to 10 sequences of amino acids, the number of elements was set to default, the maximum number of motifs was set to 7, and the optimal pattern width was fixed from 10 to 250 residues. The hidden model was generated for individual motifs occupying highly conserved topology among other species. The alignments of the seven conserved motifs were obtained from 20 different sequences of various dissimilar plants. Each model of the aligned sequences was screened across the CtCYP82G24 full-length protein sequence (Table S1). The results were represented in seven different colours, including green, yellow, pink, blue, red, grey, and light green, indicating seven conserved domains. The graphical representation of protein motifs/domains was created using the online tool EvolView v.2 (http://www.evolgenius.info/).

### 2.4. Extraction and cDNA Cloning of CtCYP82G24

To isolate the full-length cDNA sequence of *CtCYP82G24*, the flower petals of the *JH1* cultivar was used as a source of mRNA which was sampled and placed into liquid nitrogen before RNA extraction. The total RNA content was extracted using RNA Isoplus (Takara Bio Co., Beijing, China), and cDNA templates were synthesized using reverse transcriptase superscript IV (Thermo Fisher Scientific) in a reverse transcription PCR system. The amplification protocol includes the following set of gene primers. The forward primer contained the EcoRI recognition site along with a start codon for translation initiation. On the other hand, the reverse primer consists of a BamHI restriction site just after the stop codon.

CYP-24F:5′ATAGAATTCTTAATCATAGAGCTCCGAAGA3′ (62 °C)

CYP-24R:5′AATAGGATCCATGGCCGACGACTATGGC3′ (58 °C)

The primers were designed according to the Kyoto Encyclopedia of Genes and Genomes Pathway information. The exact PCR product of *CtCYP82G24* was amplified with *Pfu* DNA polymerase (Takara, Beijing, China) and then successfully cloned into the pEASY-T1 vector (Takara, Dalian, China), and the recombinant construct was further transformed into *E. coli* (TransT1) competent cells through the heat and shock method. Then, the gene of interest in the pEASY-T1 vector was sent for sequencing to observe any base mutation.

### 2.5. Subcellular Compartmentalization of CtCYP82G24

The estimated subcellular position of CtCYP82G24 was initially observed using the LocTree3 online portal (https://rostlab.org/services/loctree3/). Then, the full length of *CtCYP82G24* was amplified using the forward primer CtCYP-24FYXZ: 5′AATAACTAGTATCATAGAGCTCCGAAGA3′ containing the restriction site for BglII enzymes. The reverse primer CtCYP-24RYXZ: 5′ AATAAGATCTATGGCCGACGACTATGGC3′ contained a Spe1 recognition site. The positive bands of CtCYP82G24 were recovered from 1% agarose gel after digestion with a 200 uL double digestion system at 37 °C for 4 h. The restriction digestion pattern of CtCYP82G24 with BglII and Spe1 was analyzed on 1% agarose gel electrophoresis. After Sanger sequencing, the 1368 bp fragment of *CtCYP82G24* was fused with the linearized pCAMBIA1302-GFP-CaMV35S vector using T4 ligase. After re-confirmation by Sanger sequencing, the recombinant vector (pCAMBIA1302-CtCYP82G24-GFP-CaMV35S) was successfully transformed into *Agrobacterium tumefaciens* (EHA105 strain) following the protocol of freeze and thaw transformation. At the same time, an empty vector of pCAMBIA1302-GFP-CaMV 35S alone was also transformed into *A. tumefaciens* and was used as a control. Transformed colonies were selected on the Yeast extract peptone agar plates containing 50 mg/L of streptomycin and 50 mg/L of kanamycin; they were confirmed by the half colony PCR method. After confirmation by PCR, the single colony was resuspended in a YEP liquid medium for 24 h. Lastly, the the *Agrobacterium* culture was harvested at an OD_600_ of 1.0. The infiltration of onion epidermal layers with the suspension of *Agrobacterium* was performed for 20–30 min and then transferred to a dark room for 26 h. Transformants were selected for the observation of GFP signals in comparison with the control under a laser confocal microscope.

### 2.6. Expression and Induction of Recombinant Protein

The full-length cDNA of *CtCYP82G24* was cloned into the prokaryotic expression vector (pET28a^+^) using *Pfu* DNA polymerase (Takara). The primer pairs included CtCYP28-F (5′TTAATCATAGAGCTCCGAAGA3′) with an added EcoRI recognition site and CtCYP28-R (5′ATGGCCGACGACTATGGC3′) with an added BamHI site. The cDNA library was constructed followed by amplifying the target bands. After sequence confirmation, the double restriction digestion of *CtCYP82G24* and pET28a^+^ was performed using 200 uL plasmid digestion systems. The ligation of the *CtCYP82G24* digested product into the appropriate EcoRI, and BamHI sites of the linearized pET28a^+^ plasmid were carried out with the help of the T4 ligase. The recombinant construct (Pet28a^+^-CtCYP82G24) was inserted into the competent BL21 (*E. coli* cells). The transformed colonies were detected on an LB agar (solid) medium containing 50 mg/L of kanamycin using half colony PCR methods. Following the protocol described by [24] with slight modifications, the CtCYP82G24 recombinant protein was successfully expressed. For this purpose, the transformed BL21 cells containing pET-28a^+^-CtCYP82G24 were cultured in 500 mL LB media incubated with 50 mg/L of kanamycin. The OD of the inoculums were measured, and when the growth rate reached a maximum of 2 × 10^8^ cells/mL (A600 = 1.0), it was supplemented with 0.4 mM Isopropyl β- d-1-thiogalactopyranoside (IPTG) under an aseptic environment, and the incubation of the cells was started at 37 °C for 6 h. After incubation with IPTG, the cells were transferred to large centrifuge tubes followed by centrifugation at 12,000× *g* for 10 min at 4 °C. The cells were collected by removing the supernatants, and the remaining pellet was resuspended in a PBS buffer followed by centrifugation at 12,000× *g* for 10 min at 4 °C. A 100 mL lysis buffer composed of (12.1 g of Tris-base, 5.84 g of NaCl, and 0.58 g of EDTA) was added to harvest the cells. Pulses sonication was performed in three intervals for 15 s until soluble fractions. Bovine serum albumin and N-terminal histidine tagging were used for CtCYP82G24 protein purification with an Ni-NTA affinity column. We first washed the affinity column with the help of equilibrium buffer containing Na2HPO4 and NaCl, (20 mM:200 mM:500 mM), and then the CtCYP82G24 product was eluted using a binding buffer (20 mM imidazole). Finally, the adsorbed protein content of CtCYP82G24 was rinsed in the elution buffer, and the imidazole from the binding buffer was washed out with the help of the equilibrium buffer. After the thorough purification of Pet28a^+^-CtCYP82G24, the soluble products were separated on 12% SDS-PAGE, and the results were visualized with the help of Coomassie brilliant blue.

The quantification of metabolites were performed according to the instructions of [25]. The products were further analyzed using a high-performance liquid chromatography system equipped with a multiple-wavelength detector (MD-2010, JASCO, Japan). The quantification of samples was analyzed by the measurement of the integrated peak against two different authentic standards, including rutin and dihydrokaempferol. The data were further analyzed using the statistical significance of the differences by measuring the retention time and abundance values of three independent replicates at 415 nm UV spectra. The column temperature was kept 28 °C with a flow rate equal to 0.7 mL/min according to the previous protocol of [26].

### 2.7. Vector Construction and Agrobacterium-Mediated Transformation of Arabidopsis

The full length of *CtCYP82G24* from the flower petals of *JH1* cultivar was amplified using the CYP-24F and CYP-24R pair of primers. The CtCYP82G24 full-length coding region was ligated into the BamHI/EcoRI digested pBASTA-harboring *BAR* gene under the control of the 35S (CaMV) promoter. T4 ligase was used to ligate the gene of interest and plant over-expression vector according to [27]. The recombinant product was transformed into DH5α *E.coli*-competent cells by the heat and shock method, and the positive colonies were detected with colony PCR and confirmed by Sanger sequencing. The recombinant vector pBASTA-CtCYP82G24, as well as the pBASTA empty vector, was transformed into *A. tumefaciens* (EHA105 strain). The genetic transformation of wild type *A. thaliana* was carried out using the floral-dip infiltration method with slight changes according to [28]. T1 seeds were screen out by spraying BASTA to set T2 and T3 generations of transgenic *A. thaliana*. The PCR amplification of the *CtCYP82G24* transgene, the herbicide resistance *BAR* gene, and the *NOS* terminator gene were performed using GoTaq DNA polymerase (Promega Corp, Madison, WI, USA) and the corresponding primer pairs (Table S2).

### 2.8. Metabolites Accumulation in Transgenic Plants

We performed different elicitation experiments on transgenic plants maintained previously in a growth chamber of the Ministry of Education Engineering Research Center of Bioreactor and Pharmaceutical Development at Jilin Agricultural University. To investigate the abundance of metabolite accumulation, the homozygous T3 plants was selected at (approximately 25 days 12-leaf stage) and then incubated with PEG-induced osmotic stress (6% PEG6000) at four different time periods (0, 3, 6, and 9 h) in a Hoagland’s solution. Five individual transgenic plants were selected to reduce the possible errors obtaining from the differences of individual plants. The leaves of transgenic T3 homozygous plants were weighted precisely to a 1 g fine powder and then soaked in 14 mL distilled water–alcoholic solution for the ultrasonication extraction of the metabolites using the following conditions: Extraction temperature, 60 °C; extraction period, 30 min twice; and centrifugation at 5000 rpm for 10 min. An aliquot of 0.5 mL of sample (1 mg/mL) was mixed with 0.1 mL of 10% aluminum chloride and 0.1 mL of potassium acetate (1 M). In this mixture, 4.3 mL of 80% methanol was added to make a 5 mL volume. This mixture was vortexed, and the absorbance was spectrophotometrically measured at 415 nm. The total flavonoid content was expressed as milligrams of rutin/dihydrokaempferol equivalent (CE) per 100 g fresh weight or dry weight. Each sample was quantified using three biological replicates.

### 2.9. Quantitative RT-PCR Analysis

To further explore the *CtCYP82G24* transcript abundance in transgenic plants, five homozygous T3 lines of *A. thaliana* fresh leaves were converted to a fine powder in liquid nitrogen and then stored at −80 °C until cDNA template synthesis for qRT-PCR analysis. The relative expression level of *CtCYP82G24* in five individual transgenic T3 plants was analyzed, and the transcript abundance was measured using the 2^-△△Ct^ method. Furthermore, the expression level of *CtCYP82G24* in the selected transgenic lines under PEG-simulation at different time periods was also investigated. For this purpose, healthy plants were individually treated with PEG6000 following four incubation periods after 0, 3, 6, and 9 h. After PEG induction, the total RNA content was isolated by using RNAiso plus reagent (Takara, Beijing). The first-strand cDNA was synthesized through reverse transcription PCR using the PrimeScript RT reagent kit (Takara, Beijing) according to manufacturer’s instructions. Simultaneously, under the same conditions, the qRT-PCR assay was also investigated in ARB3-CYP24 transgenic line to determine the transcription levels of the eight core structural genes of the flavonoid pathway compare to wild-type plants. All qPCR reaction were performed in a fast real-time PCR system (Applied Biosystems 7300/7500/7500, CA, USA) using 20 μL reaction including 10 μL SYBR Premix Ex Taq (Tli RNaseH Plus), 0.3 μL ROX Reference Dye, 0.4 μL F/R primer, 1 μL template and 7.9 μL ddH_2_O. The thermal cycle includes an initial step of denaturation at 94 °C for 30 s), followed by 40 cycles at 94 °C for 30 s and final step at 60 °C for 3 s. All qRT-PCR reactions were carried out in three replicates, and the relative expression was normalized with the 18s ribosomal RNA gene (AT5G38720.1) which was used as a housekeeping gene in our analysis. The expression level was calculated according to the 2^−△△Ct^ method. The detail list of primers used for the qRT-PCR analysis of key structural genes of the flavonoid pathway in transgenic *Arabidopsis* is listed in Table S3.

### 2.10. Statistical Analysis

The data are demonstrated as mean values ± Standard Deviation. with three independent biological replicates. The each group differences between mean values were measured by a one-way analysis of variance using Statistix 8.1 software (USA). Asterisks indicate statistical significance (* *p* < 0.05, ** *p* < 0.01).

## 3. Results

### 3.1. Discovery and Functional Annotation of CtCYP82G24

The RNA-seq transcriptomic data of the *Carthamus tinctorius* cultivar *Jihong No. 1* were created using a GS-FLX sequencer (Roche, Basel, Switzerland) and then submitted to NCBI under accession number PRJNA399628. After deleting the low-quality reads, adapter reads, short and poly-N reads, a total of 577,664 and 562,930 high qualities reads from 583,440 and 567,884 raw reads were obtained in the early and full flowering, respectively. A total of 51,591 transcripts were assembled using the MIRA program [25]. The average length of the unigenes ranged within 679–5109 bp. Moreover, these unigenes were further assigned to Go terms, which were classified into a total of 43 functional groups mainly divided into three Go categories, including molecular function, cellular components, and biological processes. A high number of unigenes were assigned to biological processes followed by cellular and metabolic processes. To categorize the physiological pathways specific to *Carthamus tinctorius*, a total of 51,591 unigenes were annotated and mapped against the Kyoto Encyclopedia of Genes and Genomes (KEGG) database, resulting in 281 KEGG pathways in which 187 pathways were predicted as up-regulated and 189 as down-regulated during the metabolic process. Based on predicted open-reading frames, the unigenes were further subjected to cytochrome P450 database (http://drnelson.uthsc.edu/CytochromeP450.html). The candidate CYP450 was named according to the shared homology with reference plant (*CtCYP82G24*).

### 3.2. Phylogenetic and Conserved Domain Analysis

**Figure 1:**
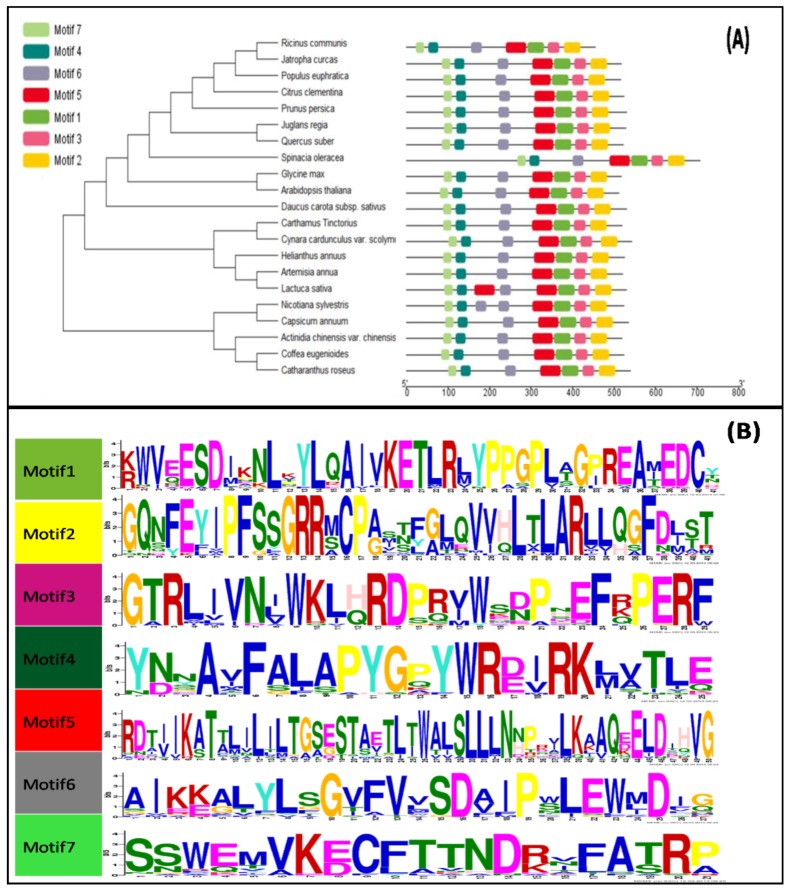
Phylogeny analysis and identification of the conserved domains (**A**) Phylogenetic analysis using the 1000 rapid bootstraps method was created with the MEGAx online tool. The online tool of the MEME server was used to identify the conserved motifs. The green, yellow, pink, blue, red, grey and light green colors represent motifs 1, 2, 3, 4, 5, 6 and 7, respectively. The indication of the grey line length denotes the sequence length. The presence of each block at multiple positions determines the location of the conserved motif to its matching one. The tree and motifs parameters were edited using the EVOLVIEW online tool (**B**) Conserved motif compositions and the representation of logos. The capital letters in the logos of individual motif represent more than 70% ratio of the conserved amino acids. The Arabic numerals appear beneath uppercase letters and indicate the width of the conserved motif.

To investigate the phylogenetic relationship, a maximum likelihood phylogenetic tree was created by adding CtCYP82G24 sequences from different species to the alignment using the 1000 rapid bootstraps method. The results revealed that the CtCYP82G24 amino acid sequence shared high similarity with homologous sequences from *Cynaracardunculus* var. *scolymus* (97.7%), *Helianthus annuus* (95.1%), *Artemisia annua* (90.6%), *Lactuca sativa* (87.3%), *Aristolochiadebilis* (85.71%), *Arabidopsis thaliana* (69.55%) and more than 60% similarity with homologous sequences from Citrus Clementina, *Glycine max*, *Catharanthus roseus* and *Quercus suber* (Figure 1A). To identify the conserved protein motifs of the CtCYP82G24 protein, we screened other CtCYP82G24-like proteins from different plant species using the MEME online tool [29]. We identified seven conserved motifs with different domain positionings (Figure 1B). Five of the conserved domains were found consistent with the presence of CG-1 and S-adenosyl-L methionine binding sites. The putative NAD(P)H-bispecific enzyme and calcium-binding domains were also observed. Altogether, the distributions of these conserved domains were identified as stress-responsive elements in *Arabidopsis* [30] and other plants [31,32,33], suggesting the importance of CtCYP82G24 to environmental stress responses.

### 3.3. Cloning and Characterization of CtCYP82G24

The full-length cDNA sequence of *CtCYP82G24* was successfully cloned from *Carthamus tinctorius* using a gene-specific primer pair (Figure S1). A 1368 bp long fragment was produced by PCR. This complete cDNA sequence contains an open reading frame encoding 455 amino acids with a 6.55 theoretical isoelectric point and 51.8 kDa predicted molecular weight. Further sequential characterization and phylogenetic analysis revealed the presence of serine/threonine conserved domain at the N-terminus. This indicates the origin of CtCYP82G24 with the oxidoreductase superfamily, which could be subject to various kinds of biochemical regulatory reactions at the cellular level. An analysis of the 3D protein model revealed certain physiological domains consistent with other plants. One of the domains contained a loop-helix grasp-like motif (Figure S2). The instability index (II) was recorded at 36.06.

### 3.4. Subcellular Localization of CtCYP82G24

**Figure 2:**
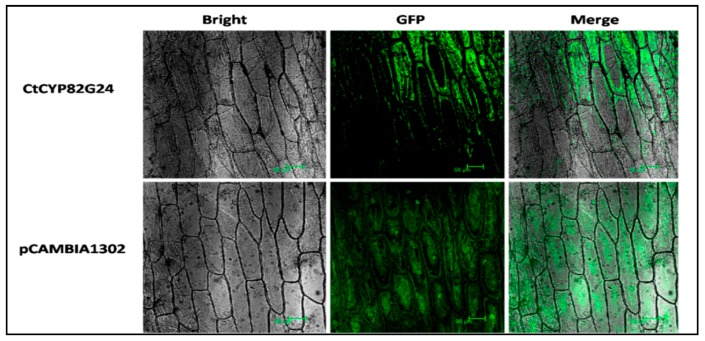
Transient transformation of CtCYP82G24. Subcellular localization of the pBASTA1302-green fluorescent protein (GFP)-CtCYP82G24 in onion epidermal cells. The fluorescence signals were detected using a confocal laser scanning microscope.

The subcellular localization of CtCYP82G24 was computationally determined using the online the LocTree3 portal. Each prediction was calculated with a confidence score (0 = unreliable and 100 = reliable) and gene ontology (GO) term of the predicted localization class. The result predicted that CtCYP82G24 might be localized to the cytoplasm or endoplasmic reticulum (Figure S3A). To experimentally determine the CtCYP82G24 localization, the complete cDNA of *CtCYP82G24* was cloned into the BglII-Spe1 digested site of a pCAMBIA-1302 vector containing a GFP driven by the cauliflower mosaic virus 35S promoter (Figure S3B). The recombinant construct (pCAMBIA1302-*CtCYP82G24*-GFP-CaMV35S) and empty pCAMBIA-1302 were efficiently transformed into the *Agrobacterium* EHA105 strain. As shown in Figure 2, the onion epidermal cells harboring the fusion construct (pCAMBIA1302-CtCYP82G24) were visualized under a confocal laser scanning microscope. The emission of GFP signals was detected primarily in the cytosol. The fluorescence of the pCAMBIA-1302 empty vector was used as a control. Our findings indicate the localization of CtCYP82G24 to cytosol, supporting previous reports on chalcone synthase and chalcone isomerase localized to cytoplasm and nuclei [34]. Chalcone synthase (CHS) and chalcone isomerase (CHI) were found to be actively involved during flavonoid biosynthesis in *Arabidopsis*. These results provided important clues to support the hypothesis that CtCYP82G24 is capable of catalysing cellular-based biological reactions in plants.

### 3.5. Heterologous Expression and In Vitro Enzymatic Activity of CtCYP82G24

**Figure 3:**
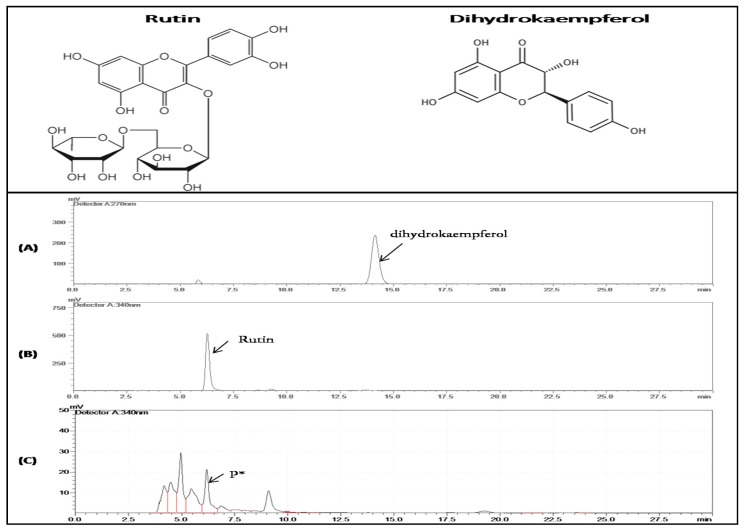
The chemical structure of rutin and dihydrokaempferol metabolites and their in vitro enzymatic activity and a high-performance liquid chromatography (HPLC) profile against the purified recombinant CtCYP82G24 protein. The protein extract was monitored at 415 nm on an Agilent Zorbax SB-C 18 column (4.6 × 150 mm, 5 μm) with methanol:acetonitrile (v:v of 1:10) as mobile phase A and 0.4% phosphoric acid as phase B. The metabolites were quantified using the aluminum chloride colorimetric method described by [35]. The total metabolite content was expressed as milligrams of rutin equivalent (CE) per 100 g dry weight (DW). (**A**) Peak labelled dihydrokaempferol and (**B**) peak marked as rutin was used as the authentic standards. (**C**) HPLC profile of the reaction product P* catalyzed by CtCYP82G24. The retention times of rutin and product P* showed close proximity in peak 4, which was approximately 6.3 min.

To confirm the function of CtCYP82G24 in vitro, we cloned the CtCYP82G24 cDNA into the expression cassettes of the pET28a^+^ vector (Figure S4) and subsequently transformed them into *E. coli* BL21 DE3 cells using heat and shock transformation. The heterologously expressed CtCYP82G24 recombinant protein was primarily detected by Coomassie blue-stained SDS-PAGE. The bands of the purified samples had molecular weights of approximately 51.8 kDa, which was consistent with the predicted size of the CtCYP82G24 protein (Figure S4A,B). The protein extract obtained from pET-28a+ alone was used as a control. The purification of the CtCYP82G24 protein was obtained using BSA and affinity chromatography (Figure S5). The in vitro enzymatic activity of CtCYP82G24 was investigated using rutin and dihydrokaempferol as substrates. Reaction products were detected by HPLC. The detection of the solvent peaks and a product P* was observed when rutin was kept as a substrate. The retention time of the product P* was approximately consistent with that of the authentic standard of rutin (Figure 3). However, the other substrate (dihydrokaempferol) was not catalyzed by CtCYP82G24, which further suggests that CtCYP82G24 may have strict substrate specificity. Additionally, the kinetic assay of CtCYP82G24 using a range of rutin concentrations showed that the V_o_ (reaction velocity) resulted in a rectangular hyperbola bar where the Vmax value was 29.9 ± 0.14 μM/min and the Km was 5.37 ± 0.123 mM (Figure S6). Our results showed the increased tendency of CtCYP82G24 corresponding to the biosynthesis of flavonoids by catalyzing the upstream precursor molecules or core intermediates of the pathway, thus revealing the partial function of CtCYP82G24.

### 3.6. Identification of CtCYP82G24 Overexpressed Transgenic Lines of Arabidopsis

**Figure 4:**
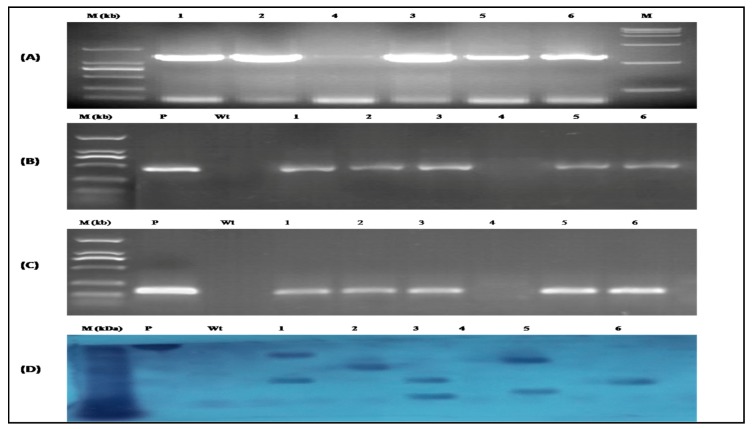
Detection of transgenic *Arabidopsis* lines harboring the pBASTA-CtCYP82G24 transgene. The growth stage for the detection was selected approximately 25 d at the 12-leaf stage. (**A**) Detection of *CtCYP82G24* (1368bp) product in transgenic plants using the CYP24R/F primer pair where M: Marker; P: Plasmid, and lanes 1–6 indicate different lines of *CtCYP82G24*—overexpressed transgenic plants. (**B**) PCR amplification of the herbicide resistance gene (*BAR*) used as a selectable marker for genetic transformation of *Arabidopsis*. WT represents the negative control, P is the recombinant vector of pBASTA-CtCYP82G24 that was used as the positive control, and lanes 1–6 indicate different lines of *CtCYP82G24* transformed plants. (**C**) Positive PCR detection of the *NOS* terminator gene in the aforesaid transgenic lines. P: Plasmid (positive control); WT: Negative control, and lanes 1–6 show the presence of the *NOS* terminator gene. (**D**) Southern blot analysis of transformed *Arabidopsis* through *BAR* probe hybridization. Where the representation was indicated as M: Marker; P: Plasmid—as positive control; Wt: Wild type—as a negative control, and lanes 1–6 represent transgenic lines.

Homozygous T3 transgenic seeds of *Arabidopsis* were grown in the growth chamber of the Ministry of Education Engineering Research Center of Bioreactor and Pharmaceutical Development at Jilin Agricultural University (Jilin, China) until the flowering stage. A selection of the transgenic plants was performed initially with by spraying (20 ug/mL) Basta solution. Healthy and viable transgenic plants were further selected for RNA extraction and cDNA synthesis by RT-PCR. The primer pair CYP-24F/R was used to amplify the full-length coding regions of *CtCYP82G24* using the cDNA of the transgenic plants as the template. Five out of six transgenic plants with a high copy number of *CtCYP82G24* were detected using 1% agarose gel electrophoresis (Figure 4A). The further identification of the transgenic plants was conducted by amplifying the herbicide resistance gene (*BAR*) to detect the presence of the pBASTA-*CtCYP82G24* transgene (Figure 4B). We also identified the nopaline synthase (*NOS*) terminator gene in the selected transgenic *Arabidopsis* plants for further confirmation (Figure 4C). The results confirmed the successful integration of *CtCYP82G24* into *A. thaliana*. We also performed Southern blot hybridization to detect the specific exogenous *CtCYP82G24* copy number. A single restriction enzyme (HindIII) digestion of the genomic DNA isolated from the transgenic plants allowed for the fragmentation of the reporter gene. As described in Figure 4D, the transformants were detected on 1% agarose gel after hybridizing with the digoxigenin (DIG)-1-dUTP probe and chemical labelling with DIG high prime. The products were immobilized by transferring them onto a nylon membrane that indicated the size of the expected BAR product together with the flanking sequence probes, thereby confirming the presence of several copy numbers of the exogenous *CtCYP82G24* within the transgenic *Arabidopsis* lines.

### 3.7. Expression Profiling of CtCYP82G24 and Metabolite Accumulation in Transgenic Plants under PEG-Induced Osmotic Stress

**Figure 5:**
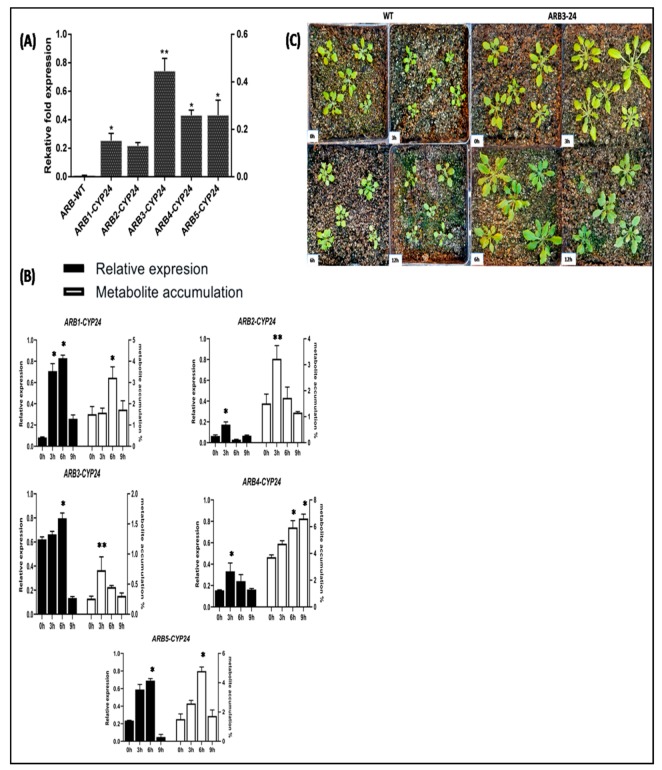
The PEG-induced expression level of *CtCYP82G24* associated with a notable metabolite accumulation in transgenic *Arabidopsis*. (**A**) The expression level of *CtCYP82G24* in transgenic lines (**B**) PEG-induced qRT- PCR assay and quantification of metabolite accumulation in *CtCYP82G24*-overexpressed transgenic plants at four different periods. Asterisks indicate statistical significance (* *p* < 0.05, ** *p* < 0.01). The relative expression level of *CtCYP82G24* was compared with that of the 18s ribosomal RNA gene (internal control). The data were calculated using the 2^−△△Ct^ method. (**C**) ARB3-24 phenotypes under PEG stress at 0, 3, 6, and 9 h intervals. The growth stage for expression and metabolite analysis was selected approximately 25 days old at 12-leaf stage.

Transgenic homozygous T3 plants were equally subjected to PEG-induction (6% PEG6000) in Hoagland’s solution for four different durations including 3, 6, and 9 h, with 0 h as the control. Then, we extracted the total RNA from the leaves of the five homozygous transgenic T3 *Arabidopsis*. The template cDNA of the individual plant was amplified using reverse transcription PCR. Each CtCYP82G24 transgenic plant demonstrated variable expression levels under various durations of simulated PEG stress. In addition, we also investigated whether the transcript levels of *CtCYP82G24* corresponded to the metabolite accumulation in transgenic plants. The qRT-PCR analysis was conducted at various times of elicitor incubation using the qRT-PCR primers of *CtCYP82G24* with 18S ribosomal RNA as the internal reference gene. The rate of metabolite aggregation was subsequently measured in the selected transgenic T3 plants. We used ARB1-CYP24 to ARB5-CYP24 as the hypothetical code for describing CtCYP82G24-overexpressed lines of *Arabidopsis* in our study. Furthermore, ARB1-CYP24, ARB2-CYP24, and ARB3-CYP24 correspond to lane 1, 2, and 3 of the previous Figure 4; however, because of the presence of transgenes in a low copy number, we followed the representation of ARB4-CYP24 and ARB5-CYP24 to lane 5 and lane 6, respectively. As shown in Figure 5, the *CtCYP82G24* transcript levels in the ARB1-CYP24 line increased at 3 and 6 h after PEG induction, whereas the accumulation of the flavonoid compounds increased over time and peaked at 6 h after PEG induction. A further comparative analysis of *CtCYP82G24* expression and flavonoid accumulation in ARB2-CYP24 line demonstrated a variable pattern under 3 h PEG induction compared to 0 h. Contrary to the transcript abundance of *CtCYP82G24* in ARB3-CYP24, the metabolite biosynthesis was increased with PEG treatment at 6 h and then decreased after 9 h, suggesting the early stress-responsive mechanism of CtCYP82G24 compared with 0 h treatment. Similarly, the result of ARB4-CYP24 and ARB5-CYP24 line indicated the up-regulation of CtCYP82G24 and peaked at 3 and 6 h, and metabolite biosynthesis was also increased gradually at these time points. However, the transcript abundance of CtCYP82G24 and metabolite aggregation declined at 9 h of PEG treatment in ARB5-CYP24 line. Based on the relative fold expression of CtCYP82G24 coupled with differential metabolite plasticity after PEG simulation, we speculate that *CtCYP82G24* transcription is up-regulated during the early phases (3 and 6 h) of PEG stress. Altogether, these results suggested that *CtCYP82G24* is an early osmotic stress-responsive gene which could promote flavonoid biosynthesis in transgenic *Arabidopsis*.

### 3.8. Overexpression of CtCYP82G24 Induced the Expression of Flavonoid Pathway Genes in Transgenic Arabidopsis

**Figure 6:**
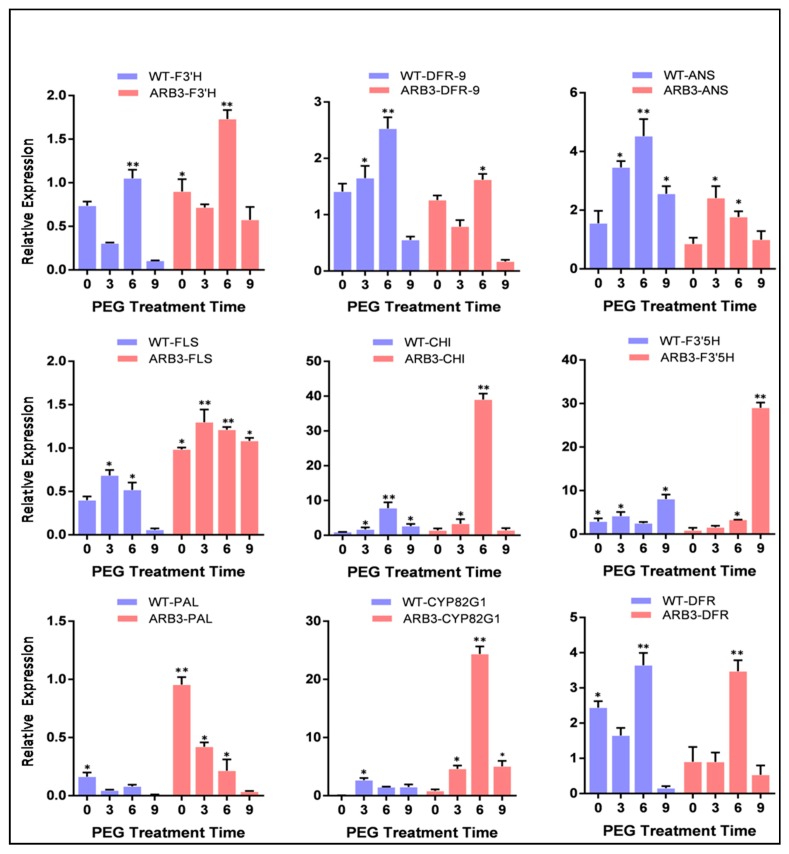
Downstream regulation of key structural genes of the flavonoid biosynthetic pathway in wild-type and *CtCYP82G24* overexpressed transgenic line. The blue colour bars represent the expression level of flavonoid biosynthetic genes in WT plants under PEG-induced osmotic stress at different time points. The red colour bars indicate quantitative RT-PCR assays of eight core flavonoid pathway genes in ARB3-transgenic under the same treatments of PEG stress. The expression levels of each transcript were expressed using the 2^−△△Ct^ method as compared to the control plants. Asterisks indicate statistical significance (* *p* < 0.05, ** *p* < 0.01). The genes were: *PAL* (*Phenylalanine ammonia lyase*), *F3′5′H* (*Flavonoid 3′,5′-hydroxylase*), *DFR* (*Dihydroflavonol 4-reductase*), *CHI* (*Chalcone isomerase*), *CYP82G1* (Cytochrome P450 *monooxygenase*), *F3′H* (*flavonoid 3′-hydroxylase*), *ANS* (*Anthocyanidin synthase*) and *FLS* (*Flavonol synthase*).

The influence of *CtCYP82G24* overexpression on the downstream regulation of key structural genes of the flavonoid biosynthetic pathway in transgenic *Arabidopsis* under PEG simulation was analyzed by a quantitative real-time PCR (qRT-PCR) assay. Our previous results demonstrated that *CtCYP82G24* was most abundantly expressed in the ARB1-CYP24, followed by ARB3-CYP24 and ARB5-CYP24 overexpression lines (Figure 5). Even so, the expression level of CtCYP82G24 was higher in the ARB1 line, and the ARB3 line showed a relatively high quality and the strongest phenotype compared to ARB1. We therefore chose the ARB3 line to investigate further insights into the role of *CtCYP82G24* in flavonoid biosynthesis and its contribution to the enhancement of the osmoregulatory mechanism. The result of the qRT-PCR assays indicated that the expression level of most of the flavonoid pathway genes, including *PAL* (*Phenylalanine ammonia lyase*), *CHI* (*Chalcone isomerase*), *CYP82G1* (Cytochrome P450 *monooxygenase*), *F3′H* (*flavonoid 3′-hydroxylase*), and *FLS* (*Flavonol synthase*) were more abundantly up-regulated in the ARB3-*CtCYP82G24* overexpression line than that of wild-type plant (Figure 6).

Nevertheless, the expression of *F3′5′H* (*Flavonoid 3′,5′-hydroxylase*), *DFR* (*Dihydroflavonol 4-reductase*), and *ANS* (*Anthocyanidin synthase*) were significantly lower than that of wild type plants. Those results may indicate that the mediation of CtCYP82G24 is involved in the flavonoid pathway by regulating the expression of flavonoid-associated genes in transgenic plants under PEG-induced stress. Similarly, the biosynthetic mechanism of the different groups of flavonoid in plants is strongly regulated by the spatial and ectopic expression of these genes, which can influence the metabolite profiling of these compounds [36,37]. Our findings also suggest that the overexpression of CtCYP82G24 could maintain intracellular osmotic balance and minimize the risk of cell membrane damage, which contributes to osmotic adjustment in transgenic *Arabidopsis*. In addition, the transcript levels of *ANS* and *FLS* showed no significant difference than the 0 h treated plants and carried the lowest expression levels under PEG induction. In agreement with our results, several copies of *FLS* and *ANS* in various plants including *Arabidopsis* have been reported that are differentially expressed in a tissue-specific manner, which controls the amount and type of flavonoid compounds [1,38,39,40,41]. Concludingly, our findings suggested that the overexpression of *CtCYP82G24* in transgenic *Arabidopsis* strongly enhances PEG-induced osmotic stress responses by regulating the transcription of key flavonoid pathway genes.

## 4. Discussion

Flowering is considered to be the most critical component in developmental processes because it ensures the growth, survival, and reproduction of flowering plants [42]. The genes in *C. tinctorius* that were expressed during four flowering stages were found to be mostly associated with flavonoid biosynthesis [43,44]. Under various stress conditions, the biosynthesis of organic compounds stimulated the defense system of plants and triggered conformational changes in biological structures that could efficiently influence the immune system of plant cells [45,46,47]. The *CYP450* supergene family has been widely investigated in many plants. Members of this family catalyze phytochemical and biosynthetic reactions that produce metabolites such as phenols, glucosinolates, phytohormones, signaling compounds, terpenoids, and flavonoids. *CYP450s* also play essential roles in regulating the homeostasis of secondary plant metabolism and hormonal crosstalk between signaling molecules [11,12]. The pharmacokinetic capacity of the *CYP450* superfamily in association with the accumulation of metabolites has been reported previously [6,7,48]. However, to date, there is no such report available on the *C. tinctorius CYP450* gene involved in osmotic stress tolerance capability by activating biosynthesis of flavonoids in transgenic *Arabidopsis*. Based on a comprehensive RNA-seq data analysis and gene annotation investigations, we aimed to assembled gene families of Jihong No.1 (JH1) cultivar of *C. tinctorius* that participate in flavonoid metabolism (data not shown). A candidate *CtCYP82G24* (*CYP450*-gene) was figured out in the KEGG database and followed by various in silico identifications (Figure 1) to speculate the importance of *CtCYP82G24* during flavonoid biosynthesis. In this study, we preliminarily identified and characterized the partial function of a stress responsive *CtCYP82G24* under the control of 35S promoter (Figure S7) underlying the molecular mechanism of enhanced flavonoid biosynthesis in transgenic *Arabidopsis*.

The subcellular studies of biosynthetic enzymes related to flavonoid biosynthesis at the cellular level have been well studied. Most were localized in cytosol and nucleus [49,50], and very few of them were localized in the vacuole [51,52] and endoplasmic reticulum [53]. Another group of enzymes was likely expressed in chloroplast and cytomembrane [54]. Flavonoid biosynthetic enzymes that are located next to plant nuclei and other cellular compartments provide DNA protectant properties against oxidative stress and UV radiation. The conversion of these genes into proteins and enzymes following isomerization and hydroxylation explained the regulation of crucial biological pathways during plant development [55,56]. Our findings implied that CtCYP82G24 was localized in the cytosol using the pCAMBIA1302-GFP fusion construct in onion cells, which is consistent with previous studies which also reported the localization of several other genes promoting flavonoid biosynthetic pathways in cytomembranes and nucleus [50,54]. Based on these results, we demonstrated the potential role of CtCYP82G24 in promoting flavonoid accumulation in *Arabidopsis*. However, the details of the regulatory mechanisms of flavonoid metabolism such as location, transport, and subsequent trafficking to numerous cellular compartments remain unclear. There seems to be a close relationship among bio-structure, transportation, and deposition of cells from flavonoids. Therefore, to fully understand the regulatory mechanisms of flavonoids, it is necessary to consider not only their metabolism but also the transportation and storage of the final product.

Combinational biosynthesis approaches such as *E. coli* and *Saccharomyces cerevisiae* host systems are widely used to produce various bioactive compounds by providing an artificial biosynthetic pathway of the gene cluster construct of an organism [57,58,59]. Different substrates that were initiated from tyrosine and phenylalanine amino acids were efficiently coexpressed using heterologous systems that could catalyze compounds such as curcumin, resveratrol, naringenin, and genistein. Additionally, *E. coli* cells incubated with precursor molecules such as carboxylic acids have produced unknown flavonoids [60]. We also investigated the in vitro enzymatic activity of *CtCYP82G24* by incorporating the PET28a-*CtCYP82G24* vector construct into *E. coli* BL21 (DE3). To confirm the in vitro function of CtCYP82G24, the purified protein was incubated with two different substrates—rutin, and dihydrokaempferol. The reaction products, which were analyzed by high-performance liquid chromatography (Figure 3), showed that CtCYP82G24 was effective in catalyzing rutin but not dihydrokaempferol. These findings are compatible with the possibility of the CtCYP82G24-induced de novo biosynthesis of flavonoids in the host system. In addition, one relatively well-known characteristic of the computational prediction of metabolite biosynthesis is to recognize metabolically reactive atomic positions in a molecule, also called the sites of metabolism (SoMs) [61]. The importance of the prediction of SoMs is crucial to know the position of a labile atom and to predict the chemical structure of metabolite where a biochemical reaction is likely to occur. In the present study, we also predicted the computational structure of rutin against a comprehensive metabolite prediction tool (GLORY) that contained the cytochrome P450 prediction module according to the instructions of [62]. The rutin metabolite structure prediction revealed that C26 is a potential site of for CYP450-induced hydroxylation (Figure S8).

Furthermore, to emphasize the importance of *CtCYP82G24* in plant stress responses, the transcript abundance and its reciprocal relationship with the accumulation of metabolites in the transgenic *Arabidopsis* under PEG-induced osmotic stress at different time points were also determined (Figure 5). The *CtCYP82G24* mRNA transcript in the ARB1-CYP24 line notably increased after 3 and 6 h of PEG induction, whereas the accumulation of the flavonoid compounds increased over time and peaked at 6 h after PEG induction when compared with 0 h. Similarly, the expression level of *CtCYP82G24* and flavonoid accumulation in ARB2-CYP24 line slightly increased at the 3 h time point compared to the 0 h control plants. Furthermore, the expression level of *CtCYP82G24* in ARB3-CYP24 and ARB4-CYP24 showed that an opposed pattern of metabolite profile was initially increased with PEG treatment at 6 h and then decreased after 9 h. These results indicate that statistically significant differences occurred at different times after PEG induction. We found that PEG pretreatment induced the up-regulation of *CtCYP82G24* up to two-to-three fold through the flavonoid dependent pathway in transgenic *Arabidopsis.* Those findings may also suggest that *CtCYP82G24* is involved in the flavonoid pathway by regulating the transcription of the core flavonoid pathway-associated genes under PEG-induced osmotic stress (Figure 6).

In a further analysis, we detected an increase antioxidant activity of CtCYP82G24 during the in vitro investigation on the presence of free radicals in CtCYP82G24 transgenic *Arabidopsis* (data not shown). The deposition of flavonoids is not limited to vacuoles and cell walls, as it also occurs in chloroplasts [63,64]. Flavonoid compounds associated with antioxidant properties have been mostly reported as the core inhibitors during auxin transport in plants [65,66]. The ability of flavonoid molecules to modulate the movement of auxin and several other cellular functions in plants [67] is because of the affinity of flavonoids to attach with the ATP binding sites of various proteins [68]. Moreover, a general stress response may cause the downregulation of photosynthetic reactions by closing the stomatal aperture in plants and turn on other unusual energy resources as an alternate mechanism [69,70,71]. Based on these reports, the concept of flavonoid-associated osmotic stress signaling originates with conceivably active antioxidant properties. Our results also speculate that the differential expression of several constitutive genes in PEG-responsive overexpression lines of *Arabidopsis* reveals other signaling systems including stomatal closure/aperture. Our results are in agreement with the findings of [72], who found that over-expression of the flavonol synthase gene (*TaFLS*) in wheat stimulates antioxidant activity by inducing flavonol biosynthesis, thus promoting the enlargement of the stomatal aperture. From here on, we also suggest that there may be an intrinsic relationship between *CtCYP82G24*-dependant flavonoid accumulation and the movement of stomatal aperture. Nonetheless, we cannot eliminate other regulatory and functional genes that could promote PEG-induced metabolite accumulation and stomatal opening in plants. Thus, more efforts are still required to clarify the effect of PEG on other functional genes involved in the flavonoid biosynthetic pathway.

## 5. Conclusions

In summary, our findings presented the discovery and characterization of a new *CtCYP82G24* isolated from *Carthamus tinctorius* using an expressed sequence homology-based approach followed by GFP tagging and expression analysis in a transgenic system. The result of the overproduced CtCYP82G24 protein during in vitro enzymatic activity is strongly associated with flavonoid biosynthesis in transgenic *Arabidopsis*. In addition, the differential gene expression level of the core structural genes of the flavonoid pathway confirmed that the mediation of *CtCYP82G24* might influence the entire flavonoid biosynthetic pathway of transgenic plants during the early phases of osmotic stress induction. These findings provide a theoretical and practical basis for identifying the intrinsic relation of a stress-responsive cytochrome P450 gene during flavonoid biosynthesis in plants.

## Supplementary Materials

The following are available online at https://www.mdpi.com/2073-4425/10/10/756/s1, Figure S1: (A) Isolation of the complete cDNA and confirmation through PCR, resulting in an exact product of (1368 bp) of ctCYP82G24. (B) Sub-cloning of the amplified product of CtCYP82G24 into the pEASY-T1 cloning vector and the detection of positive colonies through bacterial PCR using gene primers. (C) Upon successful Sanger sequencing, the double restriction digestion system was performed for the pEASY-T1 vector harboring CtCYP82G24 with BamH1 and EcoR1 restriction enzymes, Figure S2: (A) Multiple sequence alignment generated a maximum likelihood phylogenetic tree using MEGAX software, including the branch lengths and accession numbers of the proteins from different species. (B) The prediction of the CtCYP82G24 3D protein model using Phyre2 V 2.0, Figure S3: Predicted localization for the Eukarya domain of CtCYP82G24: 1. Endoplasmic Reticulum and 2. cytoplasm. The prediction confidence was measured as 18. (GO term ID: GO:0005783) (A). Confirmation of the pCAMBIA1302-CtCYP82G24 recombinant vector using double restriction digestion with Bgl11 and Spe1 restriction enzymes (B), Figure S4: (A) The cDNA cloning and identification of positive strains of BL21 harboring the CtCYP82G24 gene through PCR using the CtCYP28-F/R primers. (B) The detection of the soluble protein products of pET28a+-CtCYP82G24 separated on 12% SDS-PAGE using coomassie brilliant blue visualization where M represents the protein marker, C represents the pET28a+ control, and lanes 1–6 represent different prehydrated pET28a+–CtCYP82G24 samples, Figure S5: The standard curve of the BSA during the purification assay, Figure S6: A plot of the reaction velocity (*V*_0_) shows that the maximal velocity (*V*_max_) was approached asymptotically. The Michaelis constant (*K*_M_) is the substrate concentration yielding a velocity of *V*max/2, Figure S7: The detection of the plant overexpression vector construction (pBASTA-CtCYP82G24) using the double restriction digestion system of BamH1 and EcoR1 restriction enzymes. The schematic diagram of the plant overexpression vector (pBASTA) is given below, Figure S8: The computational structure of rutin against a comprehensive metabolite prediction tool (GLORY) that contains a cytochrome P450 prediction module, Table S1: Based on Sanger sequencing results, the user-provided protein sequence of CtCYP82G24 after translating with ProtParamExPASy, Table S2: List of primers used in our study, Table S3: List of primers used for the qRT-PCR assay of the key structural genes involved in the flavonoid pathway of the transgenic plant, Additional File 2: Calibration curve created during total flavonoid measurement, Additional File 3: Dissociation curve of the internal gene (18s ribosomal RNA) used in the q-RT PCR analysis.

## Abbreviations

GFP	Green fluorescence protein
KDa	Killo Dalton
PI	Isoelectric point
OD	Optical density
PPIase	Peptidyl-prolyl cis-trans isomerase
RRM	RNA recognition motif
ZAT	Zinc transporter family
MTPA2	Metal tolerance protein
SDS	Sodium Dodecyl Sulfate
