# Supplementary material for: Overexpression of a Novel Cytochrome P450 Promotes Flavonoid Biosynthesis and Osmotic Stress Tolerance in Transgenic Arabidopsis
Source: Genes (Basel). 2019 Sep 26;10(10):756. doi: 10.3390/genes10100756 (PMC6826380; doi:10.3390/genes10100756)

Figure S1

(**A**) Isolation of the complete cDNA and confirmation through PCR, resulting in an exact product of (1368 bp) of ctCYP82G24. (**B**) Sub-cloning of the amplified product of CtCYP82G24 into the pEASY-T1 cloning vector and the detection of positive colonies through bacterial PCR using gene primers. (**C**) Upon successful Sanger sequencing, the double restriction digestion system was performed for the pEASY-T1 vector harboring CtCYP82G24 with BamH1 and EcoR1 restriction enzymes.

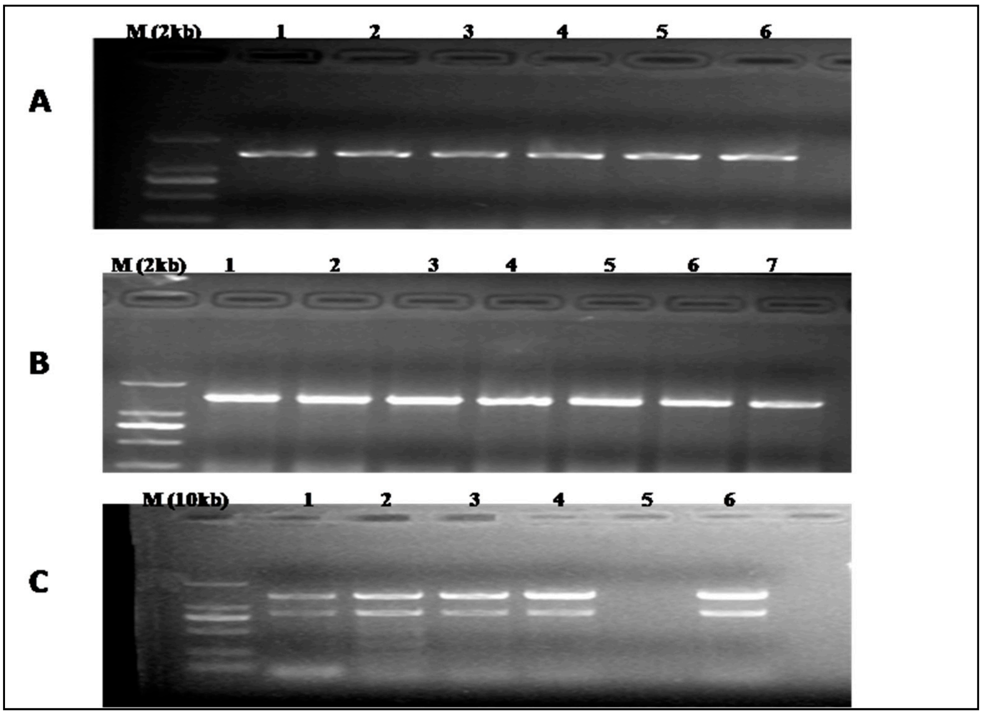

Table S1

Based on Sanger sequencing results, the user-provided protein sequence of CtCYP82G24 after translating with ProtParamExPASy.

```
10         20         30         40         50         60
MADDYGPAFS LRLGSHRAFV VSNWQMVKEC FTTNDRNFAT RPNMAVSRYM GYNQAVFALA

        70         80         90        100        110        120
PYGPYWREIR KMVTLEMLTS QRLEKLKNFR NSEVKWFVNE LFSLSASKNR DGKVEMMKRF

       130        140        150        160        170        180
ENVMFNIIVR MLAGKRFSSG GSDESGNEDL RVKEAIKKGL YLSGVFVVSD VIPSLELMDI

       190        200        210        220        230        240
GGHLKAMKQA AKELDSILEK WLDEHVEKRT EYGGDKETDF MDVMLSKLSK DAEMFSYGRD

       250        260        270        280        290        300
TIIKATTLIL ILTGSESTAE TLTWTLSLLL NTPRVLQAVQ KELDIHVGRE KWVEESDIKN

       310        320        330        340        350        360
LRYLQAVVKE TLRMYPPGPL AGPREAIEDC NIGGYHISKG TRLIVNVWKL HRDPQVWSDP

       370        380        390        400        410        420
HEFRPERFLE EHSEVNYQGQ NFEYIPFSSG RRMCPASTFA LQVVHLTLAR LLQGFDLSTP

       430        440        450
MGMPVDMSEG LGIALPKVKP LEVVIAPRLS SELYD
```

Figure S2

(**A**) Multiple sequence alignment generated a maximum likelihood phylogenetic tree using MEGAX software, including the branch lengths and accession numbers of the proteins from different species. (**B**) The prediction of the CtCYP82G24 3D protein model using Phyre^2 V 2.0.

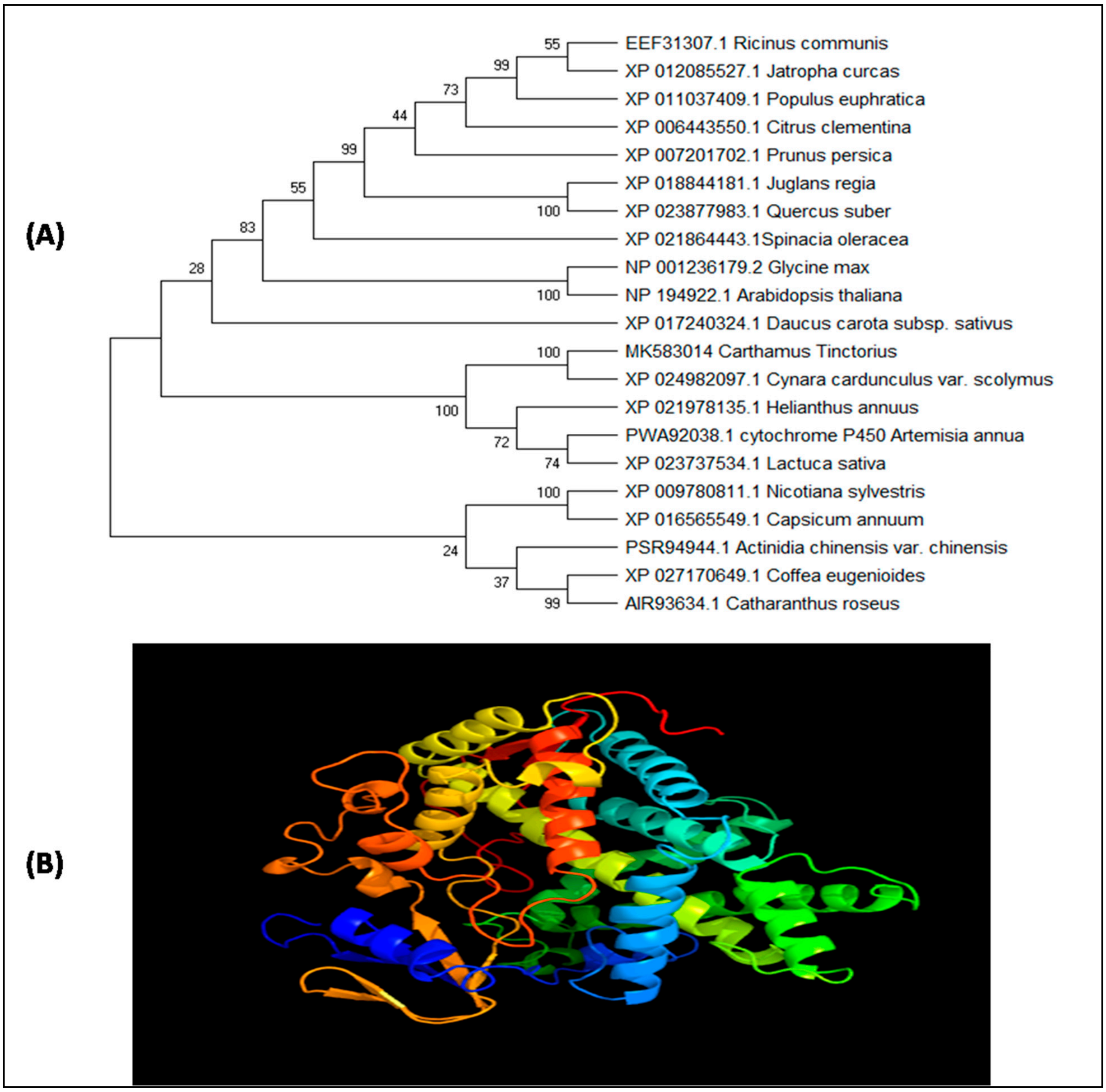

Figure S3

Predicted localization for the Eukarya domain of CtCYP82G24: 1. Endoplasmic Reticulum and 2. cytoplasm.The prediction confidence was measured as 18. (GO term ID: GO:0005783)

(A)

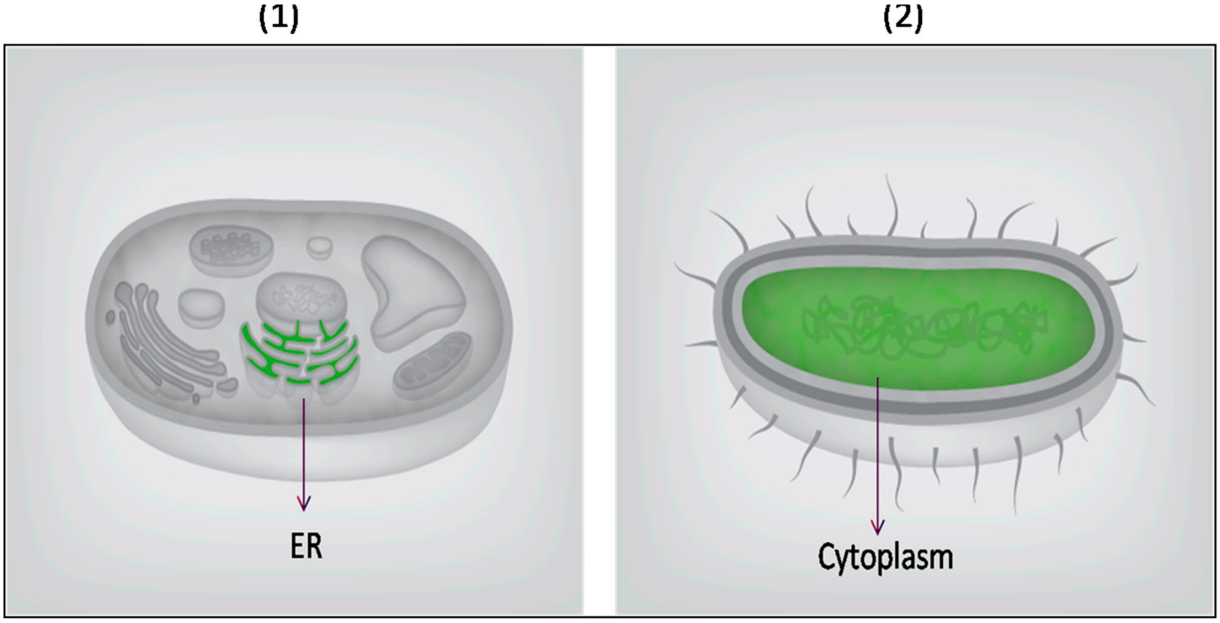

(B) Confirmation of the pCAMBIA1302-CtCYP82G24 recombinant vector using double restriction digestion with Bgl11 and Spe1 restriction enzymes.

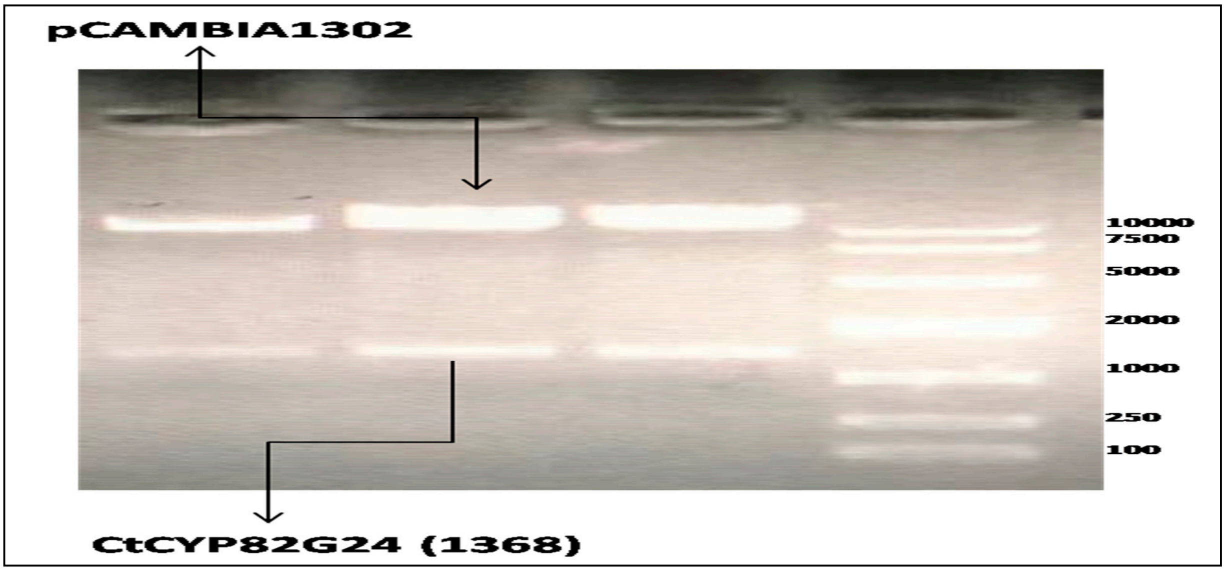

Figure S4

(**A**) The cDNA cloning and identification of positive strains of BL21 harboring the CtCYP82G24 gene through PCR using the CtCYP28-F/R primers. (**B**) The detection of the soluble protein products of pET28a$^+$-CtCYP82G24 separated on 12% SDS-PAGE using coomassie brilliant blue visualization where M represents the protein marker, C represents the pET28a$^+$ control, and lanes 1–6 represent different prehydrated pET28a$^+$–CtCYP82G24 samples.

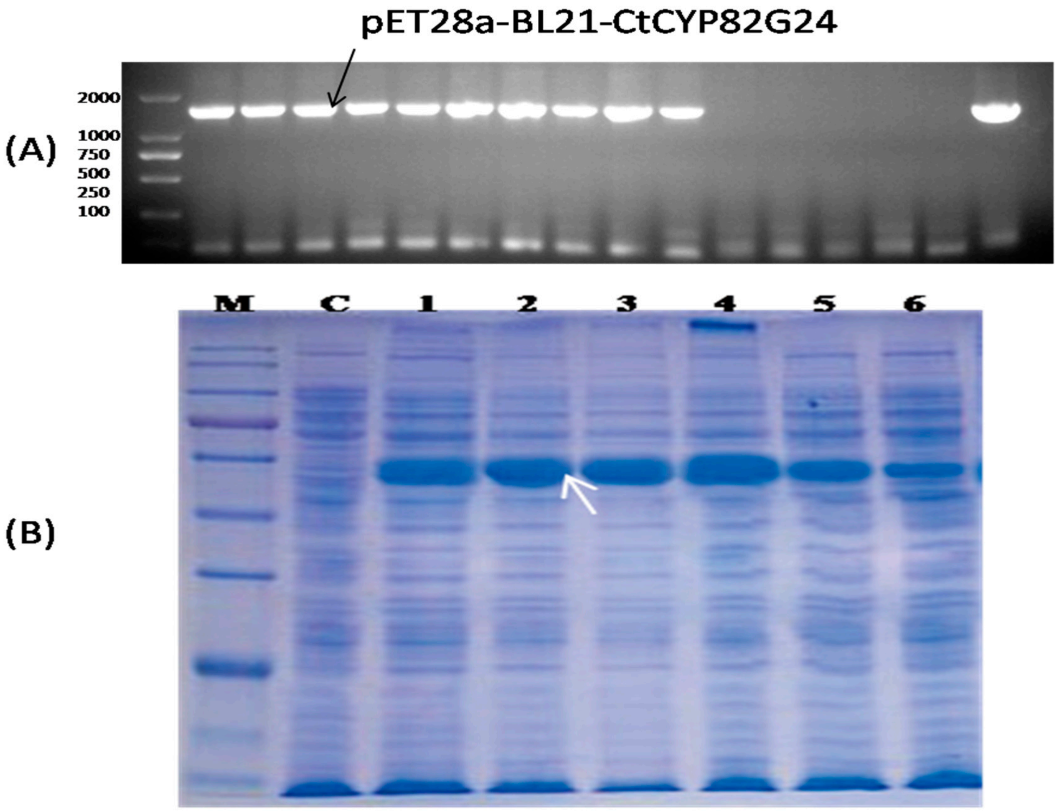

Figure S5

The standard curve of the BSA during the purification assay.

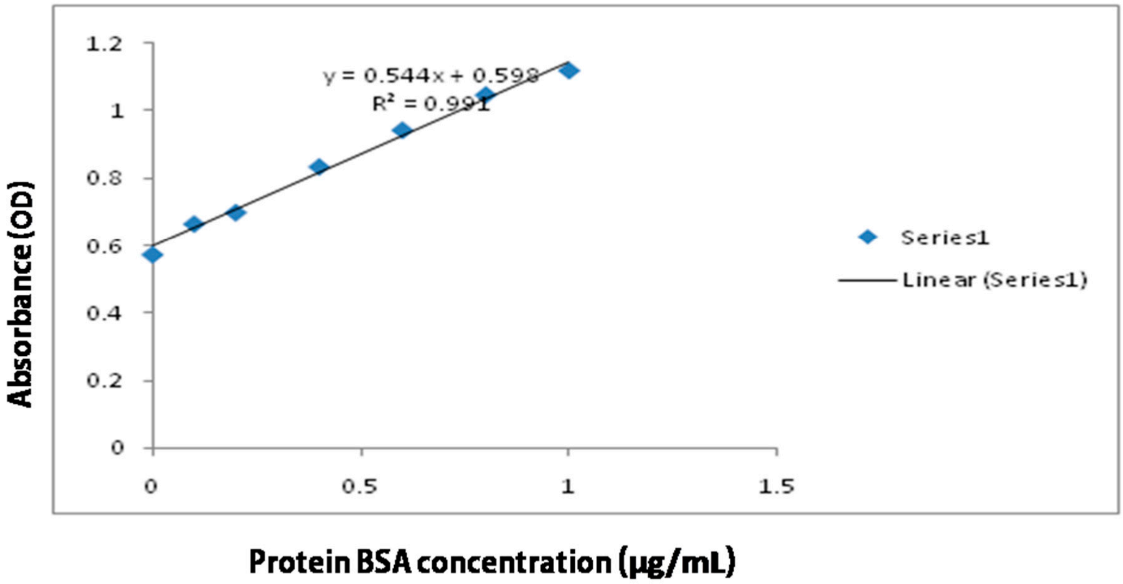

Figure S6

Michaelis–Menten Kinetics of CtCYP82G24.

A plot of the reaction velocity (V_0) shows that the maximal velocity (V_{max}) was approached asymptotically. The Michaelis constant (K_M) is the substrate concentration yielding a velocity of $V_{max}/2$.

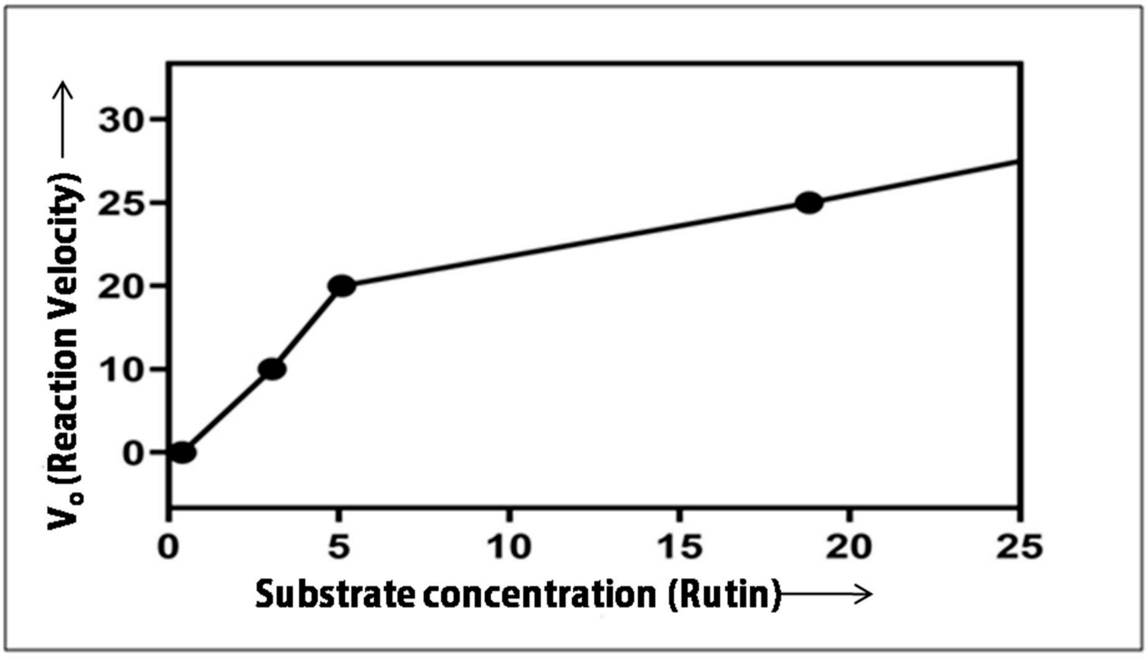

Figure S7

The detection of the plant overexpression vector construction (pBASTA-CtCYP82G24) using the double restriction digestion system of BamH1 and EcoR1 restriction enzymes. The schematic diagram of the plant overexpression vector (pBASTA) is given below.

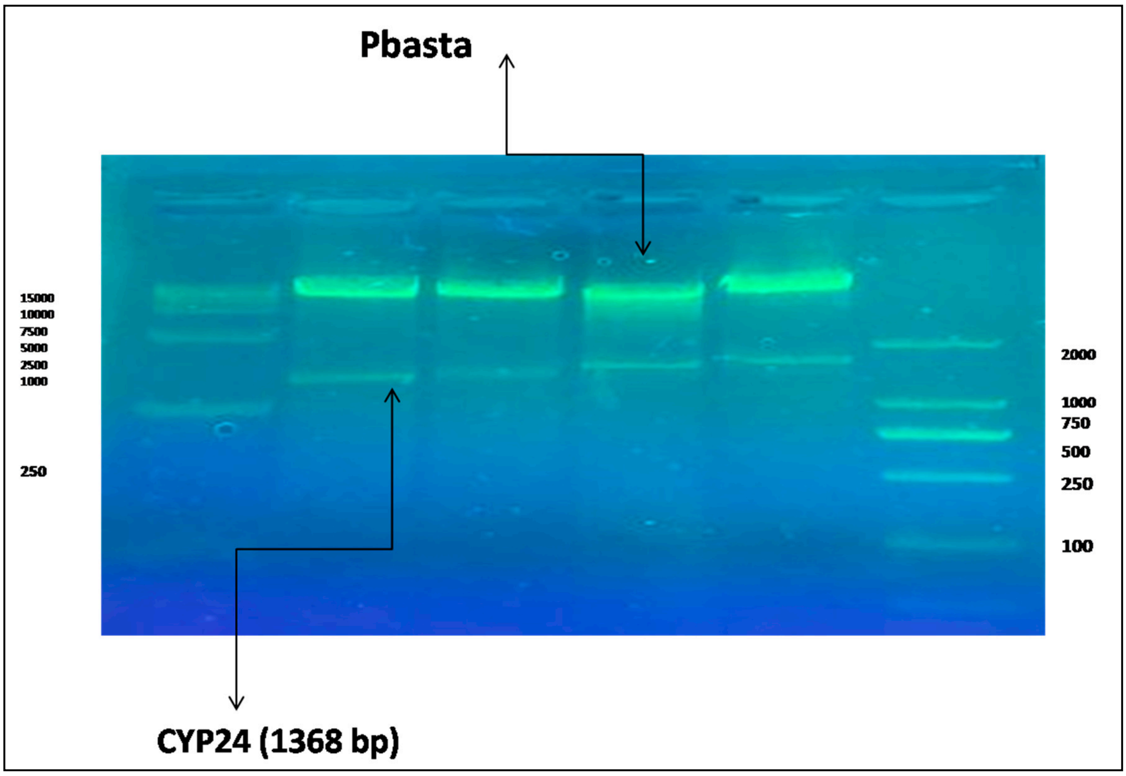

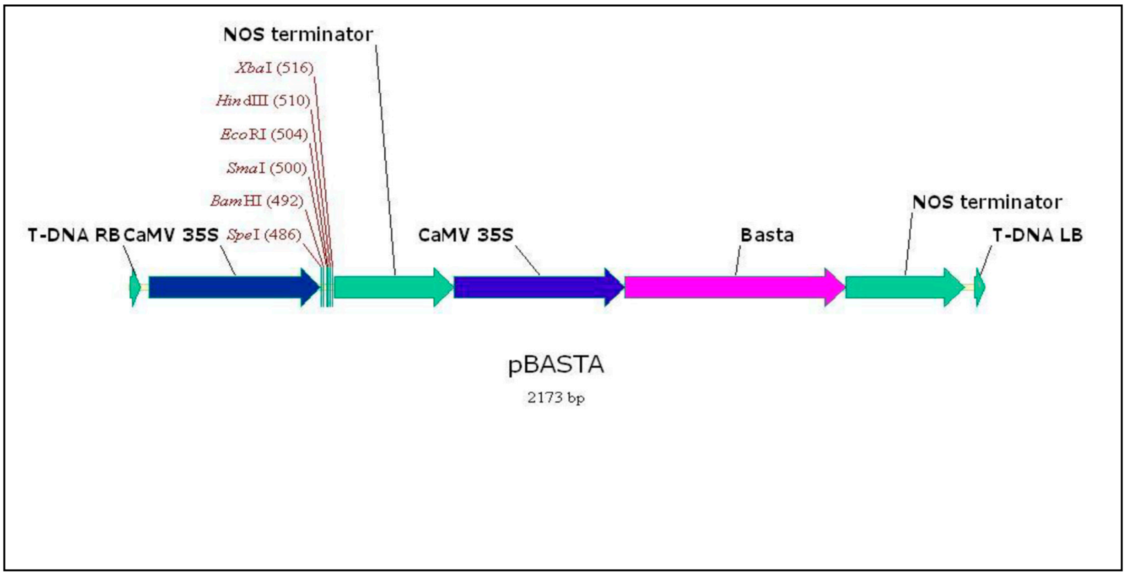

Figure S8

The computational structure of rutin against a comprehensive metabolite prediction tool (GLORY) that contains a cytochrome P450 prediction module.

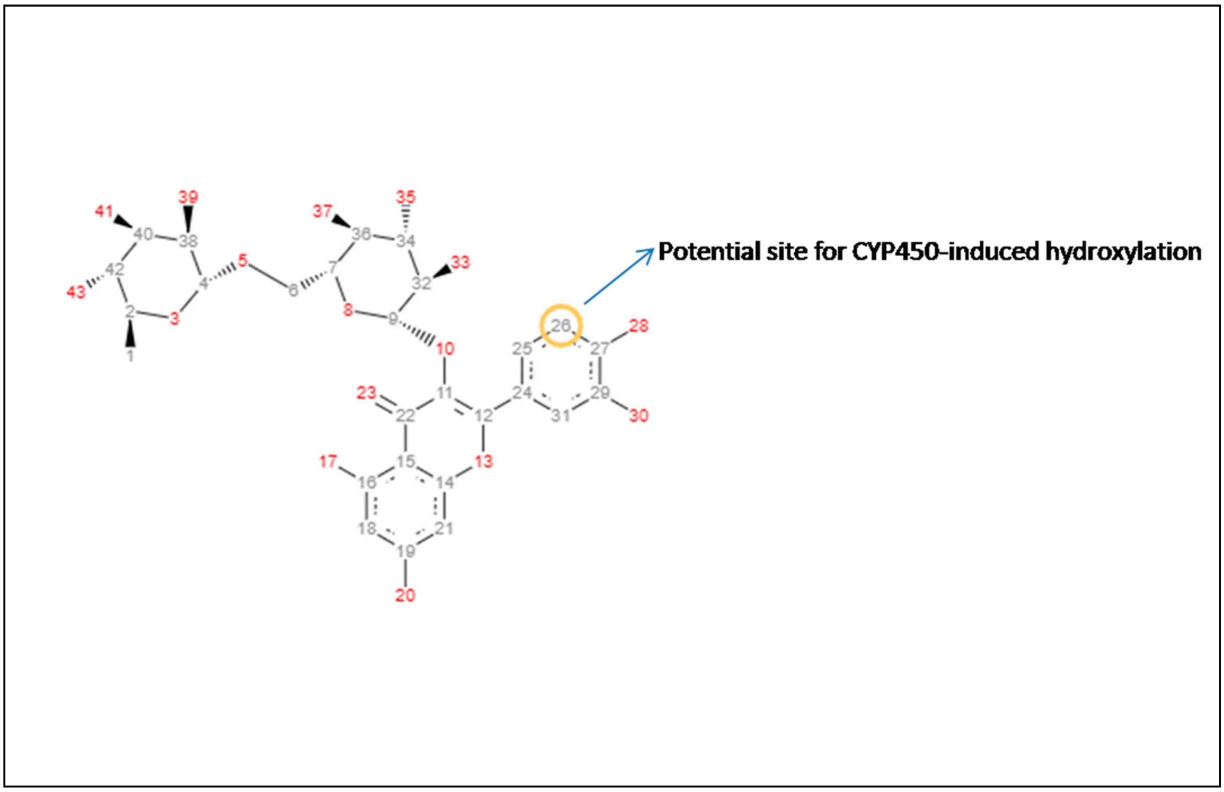

Table S2.

List of primers used in our study.

Primer code	Primer Sequences (5′-3′)	Use
**CYP-24R**<br>**CYP-24F**	AATAGGATCCATGGCCGACGACTATGGC<br>AATAGAATTCTTAATCATAGAGCTCCGAAGA	Isolation of CtCYP82G24 cDNA
**CYP-24R YX2**<br>**CYP-24F YX2**	AATAAGATCTATGGCCGACGACTATGGC<br>AATAACTAGTATCATAGAGCTCCGAAGA	Subcellular localization
**CtCYP28-R**<br>**CtCYP28-F**	ATGGCCGACGACTATGGC<br>TTAATCATAGAGCTCCGAAGA	Heterologous expression
**CtCYP82G24RT-R**<br>**CtCYP82G24RT-F**	AACGAGCCAACGGCTAGAAAA<br>TCCCGTCCCGATTCTTTGA	Fluorescence quantitative real-time PCR
**18SrRNA-R**<br>**18SrRNA-F**	TCGTTTGAGCCCGGTATTGTTA<br>GAGAAACGGCTACCACATCCAA	Housekeeping gene
**BAR-R**<br>**BAR-F**	GTCTGCACCATCGTCAACCACTA<br>TCAAATCTCGGTGACGGGC	Detection of transgenic lines
**TNOS-R**<br>**TNOS-F**	TTATCCTAGTTTGCGCGCTA<br>GAATCCTGTTGCCGGTCTTG	Detection of transgenic lines

Table S3.

List of primers used for the qRT-PCR assay of the key structural genes involved in the flavonoid pathway of the transgenic plant.

Primer code	Primer Sequences (5′-3′)	Use
**PAL1-R**<br>**PAL1-F**	GTCGACCGTCAAGAATGTGGTC<br>GCAAATCCTTTTCGCAGAAGC	qRT-PCR assay
**F3’5’H1-R**<br>**F3’5’H1-F**	AATAGGATCCCATATGATGACACCAAATTTTGGTGAT<br>AATACTGCAGTCATAAGTAGAGGTTTGTGTT	qRT-PCR assay
**DFR1-R**<br>**DFR1-F**	GTACCGAACATACATAGTTG<br>CTTCAAGTATAGTCTCGAGG	qRT-PCR assay
**CHI1-R**<br>**CHI-F**	AAGATACTTGGCAATGGTTGCG<br>CGGTATGCAACATGCCGAA	qRT-PCR assay
**CYPG1-R**<br>**CYPG1-F**	CGGACACCTCCATCTTTTGCG<br>GTGGACACGTACGCGATGTCA	qRT-PCR assay
**F3′H1-R**<br>**F3′H1-F**	GACTGAGCTAGCCGGAGAGTC<br>GAGGAGCGTGACCACAA	qRT-PCR assay
**ANS1-R**<br>**ANS1-F**	TGCCGGTGAAGGAGAAGAAA<br>CAGCCCAATCAAGCTTTTGC	qRT-PCR assay
**FLS1-R**<br>**FLS1-F**	TGAAACAAGTGGTCCACCCAC<br>ACCATAGCCAAGCCTGCAAA	qRT-PCR assay
**AT18SrRNA-R**<br>**AT18SrRNA-F**	GAAGATGAAGGAGACGAGAA<br>CTACCGTTTCCAAAGGCGAG	Housekeeping gene

Additional File 2

Calibration curve created during total flavonoid measurment

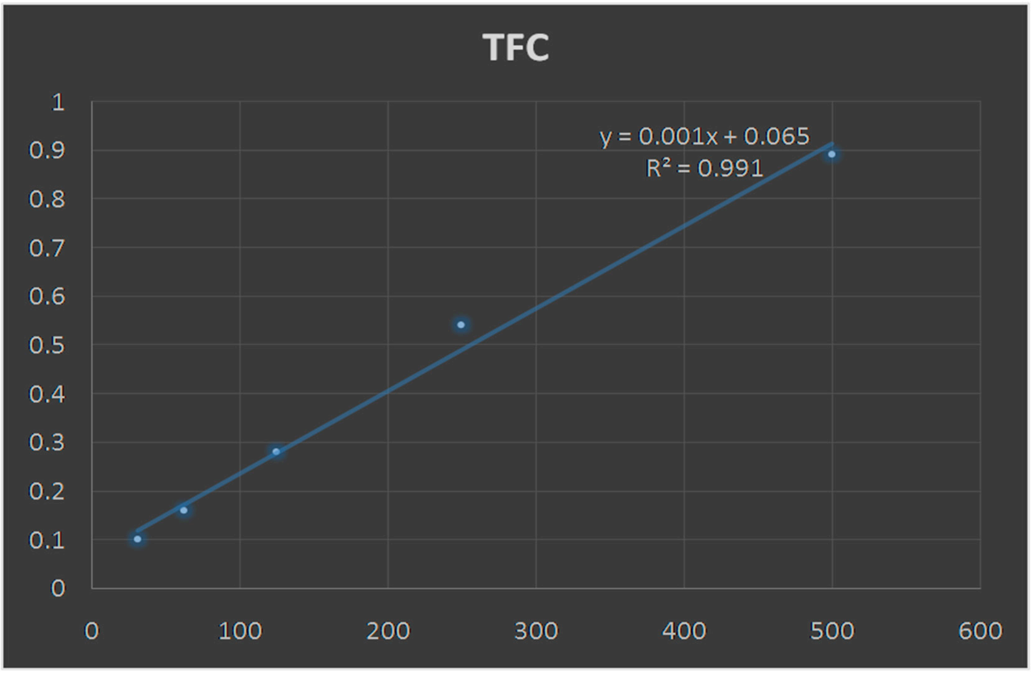

Additional File 3

Dissociation curve of the internal gene (18s ribosomal RNA) used in the q-RT PCR analysis.

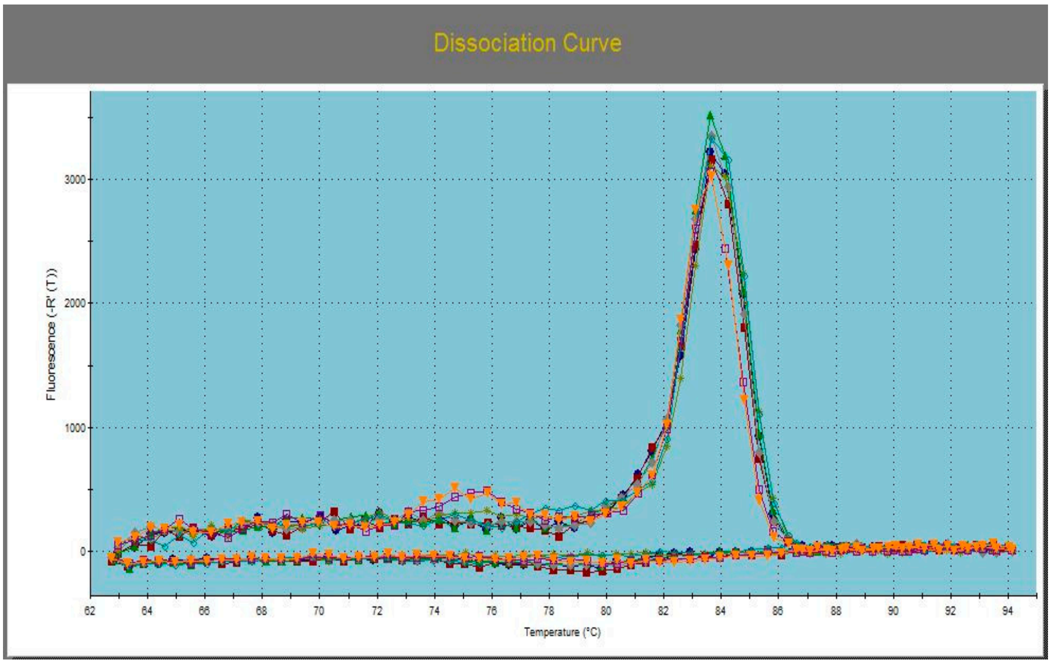

Supplement: Supplementary file 1 [file genes-10-00756-s001.pdf]
